# Nutraceutical Supplement Mitigates Insulin Resistance in Horses with a History of Insulin Dysregulation During a Challenge with a High-Starch Diet

**DOI:** 10.3390/ani14233385

**Published:** 2024-11-25

**Authors:** Caroline Loos, Annette Castelein, Eric Vanzant, Emma Adam, Kyle R. McLeod

**Affiliations:** 1 Department of Animal and Food Sciences, University of Kentucky, Lexington, KY 40546, USA; cmlo228@uky.edu (C.L.); evanzant@uky.edu (E.V.); 2 Nutrition Department, Faculty of Veterinary Medicine, Utrecht University, 3584 Utrecht, The Netherlands; 3 Gluck Equine Research Center, University of Kentucky, Lexington, KY 40503, USA; emma.adam@uky.edu

**Keywords:** horse, insulin resistance, nutraceutical

## Abstract

Insulin dysregulation (ID) is associated with an increased risk of laminitis which often necessitates the need for clinical intervention. To test the contention that the prophylactic supplementation of nutraceuticals could mitigate ID in susceptible horses, 16 mature horses with a history of ID were supplemented with either the placebo (*n* = 8) or nutraceutical (*n* = 8) once daily. Horses were housed in dry lots with ad libitum access to grass hay and fed a concentrate twice daily to provide 0.5 g starch/kg BW/meal. A combined glucose–insulin tolerance test was performed on all horses before and after 4 weeks of treatment. Nutraceutical-supplemented horses had 61% greater (*p* = 0.05) glucose clearance rates compared to the placebo group. This resulted in a shorter time in the positive phase of glucose clearance (*p* = 0.03) for the nutraceutical group compared to the placebo group. Horses receiving the nutraceutical had lower (*p* = 0.003) insulin concentrations at 75 min and lower (*p* = 0.04) glucose concentrations at 45 min compared to the placebo. Prophylactic supplementation with nutraceuticals resulted in greater glucose clearance rates during a starch challenge, indicating that nutraceuticals can mitigate ID in susceptible horses consuming an excess of non-structural carbohydrate.

## Simple Summary

Insulin dysregulation (ID) is associated with an increased risk of laminitis which often necessitates the need for clinical intervention or euthanasia in severe cases. The prophylactic supplementation of nutraceuticals could mitigate ID in susceptible horses, improve horse wellbeing, and reduce the incidence and (or) severity of this debilitating disease. Accordingly, this study was conducted to test the hypothesis that nutraceutical supplementation containing a mixture of omega-3 fatty acid sources, glutamine, vitamin E, and an active yeast mitigates insulin resistance in horses with a prior history of ID during a four-week challenge with a high-starch diet. A combined glucose–insulin tolerance test was used to assess the insulin sensitivity in each horse. After four weeks of treatment, horses supplemented with the nutraceutical had 61% greater blood glucose clearance rates and a reduced time necessary to return to the baseline concentration following a glucose–insulin bolus compared to placebo-treated horses. Likewise, plasma insulin concentrations returned to baseline concentrations at 75 min post-bolus for nutraceutical-treated horse, whilst those supplemented with the placebo remained in a hyperinsulinemic state. Thus, it was concluded that (1) the dietary high-starch challenge used in this study was sufficient to reduce the insulin sensitivity in ID-prone horses and (2) the prophylactic supplementation of the nutraceutical blend mitigated the negative effects of the dietary starch challenge on insulin sensitivity ID in susceptible horses.

## 1. Introduction

Laminitis remains one of the most complex and frustrating clinical conditions in equine veterinary practice. One report indicates that up to 15% of horses develop this debilitating condition in their lifetime and 75% of hospitalized cases ultimately require euthanasia [1]. This makes laminitis not only an important animal welfare issue, but it is also a substantial economic burden on horse owners and the equine industry. Insulin resistance (IR) has received considerable attention due to its relationship with endocrinopathic laminitis [2,3].

Of acute laminitic cases, approximately 90% are associated with underlying endocrinopathies, with hyperinsulinemia being the unifying associated factor [1,4,5]. Although hyperinsulinemia can occur in the absence of tissue IR [6], it is well-known that decreased peripheral insulin sensitivity is a major culprit for compensatory insulin release.

Insulin resistance is defined as reduced tissue insulin sensitivity leading to a decreased responsiveness to insulin-mediated glucose disposal and compensatory hyperinsulinemia in an attempt to maintain euglycemia [3,7]. Elevated circulating insulin levels can impact the integrity of the lamellar tissues in the hoof through interference with intracellular signaling and the activation of pathways that trigger a cascade of metabolic, cytomorphological, and vascular disturbances [8,9,10,11]. This pathological relationship underscores the importance of managing IR to mitigate the risk of laminitis.

While a genetic predisposition contributes to the development of IR, environmental and dietary factors are pivotal in precipitating IR. The chronic intake of high levels of non-structural carbohydrates (NSC) from concentrates or forages seems to be the main driver behind the expression of the IR phenotype [12,13,14,15,16]. Repeated bouts of hyperinsulinemia have shown to decrease the expression of glucose and lipid transporters as well as insulin receptors in various tissues which promotes an IR state [17]. Recent data suggest that a threshold of 0.1 g of NSC/kg BW/meal is prudent to avoid hyperinsulinemia in insulin-dysregulated (ID) horses [18]. This quantity is readily exceeded in most commercial feeds. Avoiding an excess NSC intake is even more difficult with forage, especially when horses are housed on pasture. Common cool-season grasses or hay can contain 10% to 20% NSC, which means that even at the lowest concentrations, with a conservative estimated intake of 1.5% BW/d, this would equate to approximately 750 g of NSC intake per day in a 500 kg horse [19].

Given these dietary challenges, prophylactic intervention strategies that may mitigate the development of IR are of intense interest to veterinarians and horse owners. A variety of nutritional compounds that may have therapeutic effects on IR have been investigated in humans and horses [20,21,22]. In particular supplementation with omega-3 fatty acids, vitamins, specific amino acids, chromium-yeast, and prebiotic compounds have shown to have beneficial effects on glucose and insulin dynamics [22,23,24,25,26]. The suggested mechanisms by which these ingredients exert their effects include improving insulin receptor signaling, cellular glucose uptake, and cellular energy metabolism, decreasing hepatic glucose production and the upregulation of PPAR-ɣ expression, improving endocrine and immune signaling, influencing intestinal microbiota and barrier function, and reducing oxidative stress [27,28,29,30,31,32,33]. Most of the previous work in horses has focused on the effect of nutraceuticals on the insulin response to oral glucose. However, there is a paucity of data looking at the potential therapeutic effect on tissue insulin sensitivity specifically. Therefore, the objective of the current study was to evaluate the effectiveness of a nutraceutical supplement in an IR-challenge model using horses with a history of ID receiving a starch-based diet.

## 2. Materials and Methods

### 2.1. Ethics Statement

This experiment was conducted at the University of Kentucky, Department of Veterinary Science Maine Chance Equine Health Research Center in Lexington, KY 40511, USA. All methods were approved by the Institution Animal Care and Use committee at the University of Kentucky (approval #2022-4053; approval date 22 April 2022).

### 2.2. Animals, Diets, and Housing

In order to evaluate the potential protective effects of the nutraceutical on development of IR in a NSC challenge model, horses did not need to have IR at the start of the study, but had to have a history or predisposition to ID. Inclusion criteria were based on regular screening data from oral sugar tests (OST) and/or CGIT results from previous research trials as well as clinical appearance and health records (i.e., BCS and previous laminitic episodes). A routine screening two months prior to the start of this study showed that 13 out of the 16 horses were hyperinsulinemic (insulin concentrations > 45 µIU/mL 60 min post oral sugar administration. Additionally, all horses showed one or more of the typical clinical symptoms of equine metabolic syndrome: (i) they were considered overweight (BCS > 6/9), (ii) they consistently had ID throughout the years as, assessed by OST or CGIT, and (iii) several of the horses also had a history of laminitis based on clinical records. None of the horses were laminitic at the start of this study or had been in the months prior. Based on basal ACTH concentrations and evaluation of clinical symptoms, none of the horses in this study were considered to have PPID (Pituitary Pars Intermedia Dysfunction, sampling was conducted in July). Following these criteria, 16 mature horses (mixed breeds and sexes, aged 16.7 ± 3.7 years old) were selected from the University of Kentucky, Department of Veterinary Science herds. Horses were housed in 2 adjacent dry lot areas (*n* = 8 per pen) with ad libitum grass hay, water, and salt and mineral blocks. Estimating a minimum of 1.5% BW in voluntary hay intake per day, each horse was individually fed 1.65 g/kg BW of a commercial high-starch concentrate (StaminOats, Hallway Feeds, Lexington, KY, USA; Table 1) twice daily to provide ~115% of daily digestible energy (DE) requirements. This amount of concentrate provided 0.512 g starch/kg BW/meal (as fed) and 0.6 g NSC/kg BW/meal, and was considered a “high starch challenge” diet for ID-prone horses (NSC calculated as starch + ethanol soluble carbohydrates). The dietary regimen was designed to meet or exceed all nutritional requirements for mature horses with ‘average’ maintenance requirements [34]. No grain refusals were recorded throughout this study. All horses were adapted to housing and dietary regimens for at least 2 weeks prior to any sample collections.

### 2.3. Experimental Procedures

In a randomized complete block design, insulin sensitivity was assessed by a combined glucose–insulin tolerance test (CGIT) before and after 4 weeks of supplementation with the nutraceutical pellets, details on methodology to follow, based on the delta glucose concentrations at 45 min (i.e., glucose concentrations at 45 min minus those at baseline) from the initial CGIT (Table 2). Horses were stratified according to their degree of insulin sensitivity and then randomly assigned to treatment groups (*n* = 8 nutraceutical, *n* = 8 placebo). In addition to equal distribution of insulin sensitivity, treatment groups were also balanced for age and body condition score (BSC). Additionally, horses were blocked by pen (*n* = 4/treatment) and the initiation of treatment period was separated by one day between blocks so that all measures were made on the same day of treatment. Treatments consisted of a (i) pelleted proprietary nutraceutical supplement (Cooperative Research Farms, Richmond, VA, USA) containing a blend of marine-derived omega-3 fatty acid sources (eicosapentaenoic acid and docosahexaenoic acid), glutamine, vitamin E, and active brewer’s yeast or (ii) placebo pellet made in the same way without the active ingredients. The carrier for both treatment pellets consisted of wheat middlings and soy hulls. Treatment pellets were fed at the manufacturer’s recommended rate (0.75 g/kg BW/day) once a day together with the concentrate feed. Body condition score (BCS) was determined by 2 independent, experienced scorers according to the scale developed by Henneke et al. [35]. Body weight was recorded weekly using an electronic scale. Horses were monitored daily, and forelimb digital pulses (DP) were evaluated twice weekly by 2 trained individuals. Digital pulses were scored on a 0–2 scale, where a score of 0 was defined as no DP, 1 as a weakly palpable DP, and 2 as a bounding DP. If a DP score of 2 was detected, lameness was assessed using the Modified Obel Grade lameness scoring method, as described by Meier et al. [36].

#### Combined Glucose–Insulin Tolerance Test (CGIT)

All horses underwent a CGIT prior to the start of the feeding trial and after 4 weeks of supplementation, as previously described [37]. On the day of CGIT, concentrate feed and hay were withheld, but access to water was maintained for all horses. Briefly, intravenous catheters were aseptically placed in the jugular vein. After a 30 min recovery period, 2 baseline blood samples were collected at −15 and 0 min. Whole blood glucose levels were measured immediately using a point of care glucometer. A sterile 50% dextrose solution (providing 150 mg glucose/kg BW) was rapidly administered intravenously (<1 min), followed immediately by insulin (0.1 U/kg BW) diluted in 3 mL sterile 0.9% saline solution. Additional blood samples were then collected at 1, 5, 15, 25, 35, 45, 60, 75, 90, 105, 120, 135, and 150 min following insulin administration. For each sample time, blood glucose concentrations were immediately determined as described above. No horses showed concerning signs of hypoglycemia or had a blood glucose level <29 mg/dL for a single 15 min time point. All blood samples were immediately stored on ice prior to centrifugation (1500× *g* for 10 min). Plasma was stored at −20 °C prior to batch analysis.

### 2.4. Sample Analyses

#### Whole Blood Glucose and Plasma Insulin Concentrations

Whole blood glucose concentrations were determined at the time of blood draw using a handheld point of care glucometer (Accu-Chek Aviva, Roche Diagnostic, Indianapolis, IN, USA). Insulin concentrations were determined from frozen plasma in duplicate using a radioimmunoassay kit (Porcine Insulin RIA, PI-12K, Millipore Sigma, Burlington, MA, USA) previously validated in horses [38]. Intra-assay variation for insulin was 6.1%.

### 2.5. Data Analysis

#### Analysis of CGIT Results

Analysis of the CGIT results was conducted as previously described [39]. Briefly, “positive phase” is defined as the phase during which blood glucose concentrations are above baseline (t = 0 min) concentrations, excluding the recovery phase (i.e., phase during which glucose concentrations exceed baseline after reaching nadir concentrations). The positive phase “duration” is defined as the time in the positive phase and calculated as the time in minutes between t = 0 min (start of CGIT) and the timepoint where the glucose concentration returned to baseline levels. Time to nadir is defined as the time between t = 0 min and the timepoint of the lowest measured glucose concentration during the entire procedure. Positive-phase glucose clearance was calculated by dividing the difference between peak and nadir positive-phase glucose concentrations by the difference between positive-phase duration and time to peak glucose concentrations. The area under the curve (AUC) for the blood glucose concentration over time was determined for the positive phase. The negative phase (time during which glucose concentrations were below baseline) and the recovery phase were not taken into consideration since it is not possible to differentiate between glucose clearance and hepatic glucose production. Delta (Δ45) t = 45 was defined as the difference between glucose concentrations at t = 45 and t = 0 min. Horses were considered insulin resistant when glucose concentrations did not return to normal baseline concentrations at t = 45 min and/or plasma insulin concentrations were >20 μIU/mL at t = 75 min (33).

### 2.6. Statistical Analysis

All data were analyzed using mixed procedures in SAS 9.4 statistical software (SAS Institute, Cary, NC, USA). Treatments were blinded for all statistical analyses. Area under the glucose response curves were calculated using the trapezoidal method with commercially available software (GraphPad Prism). A power analysis was conducted for a randomized complete block design using previous measures of insulin sensitivity within our laboratory. Using a coefficient of variation of 20%, with alpha and beta values set at 0.05 and 0.80, respectively, 8 animals per treatment were required to detect a 40% difference in measures of insulin sensitivity.

A covariate analysis was conducted on CGIT data using pre-treatment data as the covariate. The covariate independency from treatment was tested prior to analysis (*p* ≥ 0.61). Treatment (i.e., placebo or nutraceutical) was considered a fixed effect and pen (dry lot number) was a random factor in the model. Bodyweight and BCS were analyzed with a one-way ANOVA for the initial measures and the change over time (i.e., Δpre/post). Treatment was considered a fixed effect with pen (dry lot number) as random factor. Age was analyzed using a one-way ANOVA with treatment as a fixed effect and pen as random factor. A repeated measures ANOVA was performed on the digital pulse scores. Week was considered the repeated measure with horse as subject. Treatment, week, and their interaction was considered a fixed effect and pen as a random effect. Studentized residuals for all variables were evaluated graphically using Quantile–Quantile plots and with SAS univariate procedures to ensure that normality assumptions were met. Non-normally distributed data (basal insulin concentrations and time in positive phase) were log-transformed for analysis. Normally distributed data are presented as least square means and standard error of the mean unless otherwise indicated. Transformed data are presented as back-transformed data ± 95% confidence intervals. Statistical significance was considered at *p* ≤ 0.05 and trends were considered when 0.05 ≤ *p* ≤ 0.10.

## 3. Results

### 3.1. Body Weight, Body Condition Score, and Age

Body weight increased slightly in all horses throughout the study, but was unaffected by the treatment (*p* = 0.65, Table 2). The nutraceutical group showed a tendency (*p* = 0.08) to have a higher body weight at the start of the study. This is likely due to the large variation in the size of the horses and the prioritization of blocking for the insulin status rather than body weight across treatments. There was no difference in BCS between treatment groups at the start of the study (*p* = 0.41) or BCS change over time (*p* = 0.27, Table 2). There was no difference in age between the treatment groups (*p* = 0.13, Table 2).

### 3.2. Digital Pulse Scores

There was no effect of the treatment on (*p* = 0.85) the DP scores (Table 3). There was an effect of time (*p* = 0.006) where the DP scores in week 1 were lower (*p* ≤ 0.004) compared with W2 and W3 and tended (*p* = 0.06) to be lower compared with W4. There was no interaction (*p* = 0.32) between the treatment and time (Table 3). For three horses, a DP score of 2 was recorded during weeks 2 and 3. For two of these horses, a hoof abscess was found upon further assessment. The third horse underwent a lameness exam, but was not diagnosed as being lame and the DP went back down at the next recording.

### 3.3. Combined Glucose Insulin Tolerance Test

**Figure 1:**
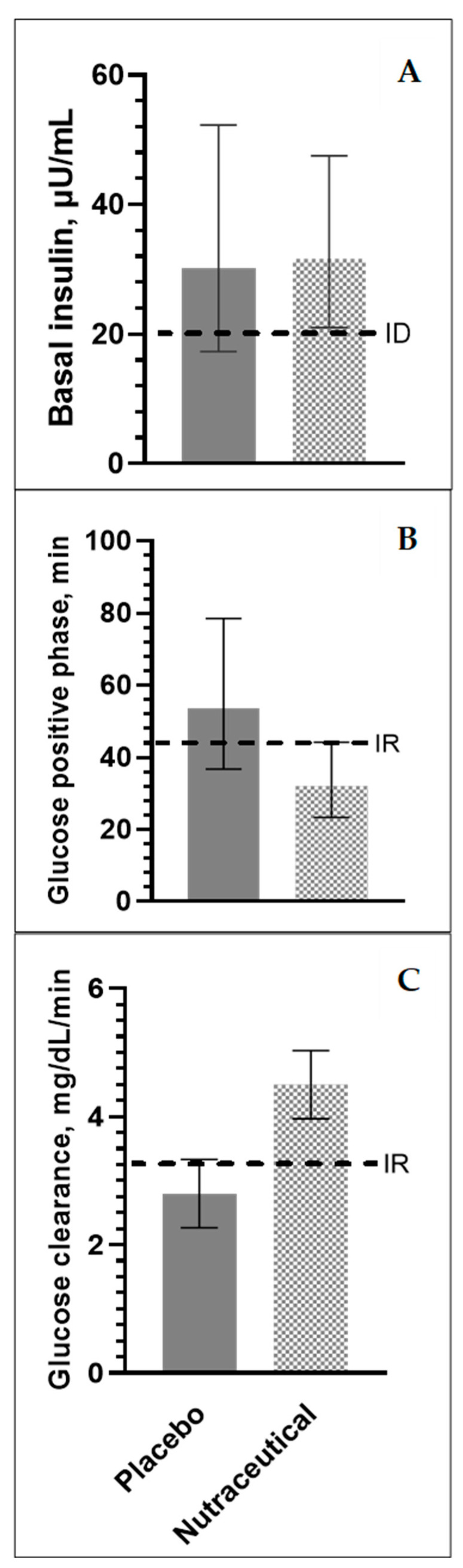
Nutraceutical supplementation and threshold indices for ID and IR in ID susceptible horses during a high NSC challenge. Plasma glucose and insulin responses during the CGIT after 4 weeks of supplementation with either a placebo (*n* = 8) or nutraceutical (*n* = 8) supplement. IR: insulin resistance. ID: insulin dysregulation. (**Panel A**) Basal plasma insulin concentrations (µIU/mL) in the placebo (solid) and nutraceutical (dotted) group after 4 weeks of treatment. Concentrations were log transformed, data are back transformed means ± 95% CI (Treatment effect, *p* = 0.90). The dotted line indicates the common threshold value (>20µU/mL) used for defining horses as having suspected ID [40]). (**Panel B**) Time in the positive phase (min.) of the glucose response curve in the placebo (solid) and nutraceutical (dotted) group after 4 weeks of treatment. Times were log transformed, data are back-transformed means ± 95% CI (Treatment effect, *p* = 0.03). The dotted line indicates the common threshold value (>45-min) used for defining horses as having IR [37]). (**Panel C**) Glucose clearance rates (mg/dL/min) in the placebo (solid) and nutraceutical (dotted) group after 4 weeks of treatment. Data are presented as least square means ± standard error of the mean (*p* = 0.05). The dotted line indicates our threshold value (<3.3 mg/dL/min) used for defining horses as having IR based on previous work [39].

After 4 weeks of supplementation, there was no difference between the treatment groups for the basal glucose (*p* = 0.17) and insulin (*p* = 0.90) concentrations (Table 4, Figure 1). However, horses receiving the nutraceutical supplement had significantly lower glucose concentrations at t = 45 min (*p* = 0.04) and, consequently, larger Δ45 glucose concentrations (*p* = 0.05) compared to the placebo group. Insulin concentrations at t = 75 min were lower (*p* = 0.003) in the nutraceutical-treated group compared to the placebo group (Table 4). Furthermore, glucose clearance rates in the positive phase were greater (*p* = 0.05, Table 4, Figure 1) in horses that had received the nutraceutical supplement compared to the placebo, resulting in a shorter (*p* = 0.03, Table 4, Figure 1) time in the positive phase and a faster (*p* = 0.03) time to reach nadir glucose concentrations. There were no effects of the treatment on peak glucose concentrations and positive-phase glucose AUC (Table 4).

## 4. Discussion

### 4.1. Laminitis and Insulin Dysregulation

Laminitis is a multifaceted but severely debilitating disease in horses [41]. Pain, problems with mobility, the recurrence of painful episodes, and poor outcomes make it a significant problem for equine welfare, horse owners, and veterinarians. Laminitis is broken down into three general categories according to the cause: endocrinopathic laminitis (including pasture-associated or hyperinsulinemic-associated laminitis), laminitis caused by systemic inflammation (i.e., sepsis-related laminitis), and support-limb laminitis. Of all cases, endocrinopathic laminitis is the most common and thought to be responsible for up to 90% of clinical laminitis [5]. Endocrinopathic laminitis is associated with insulin dysregulation (ID), a metabolic condition which is characterized by transient or persistent hyperinsulinemia, which in many cases is driven by tissue insulin resistance (IR). The mechanisms underlying this condition are complex and still not entirely understood, but prolonged exposure to circulating insulin in both in vivo and in vitro models has consistently caused damage to the structural integrity of equine lamellar tissues under hyperinsulinemic conditions [42,43]. Whether this is a direct or indirect effect on cellular cytoskeletal systems is still poorly understood and perplexing considering there are very few insulin receptors in the digital lamellae [44]. More recent theories suggest that the damaging effect of insulin may be mediated through the IGF-1 receptor and the subsequent activation of the mitogen-activated protein kinase (MAPK) and PI3K/Akt pathways, leading to cytomorphological alterations and vascular dysfunction [11,45,46]. Evidence as to whether ID contributes to the systemic or local production of pro-inflammatory cytokines is equivocal at present [47,48,49]. However, there is evidence that ID horses exhibit a prolonged expression of pro-inflammatory cytokines following endotoxin exposure [50], suggesting that ID is not only causative in endocrinopathic laminitis, but could contribute to the severity of sepsis-related laminitis.

Although genetic predisposition and obesity are significant contributors to ID, the excessive consumption of non-structural carbohydrates (NSC) is the primary contributory factor [7,13]. Modern equine diets typically contain substantial amounts of NSC provided through both commercial concentrates as well as forages. Pasture-associated laminitis continues to be a prevalent clinical condition seen in the spring and summer months when sugar levels in grazing forage tend to rise in many areas of the northern hemisphere. Controlling the NSC intake in horses at risk of laminitis is a challenging task. The findings in this study provide evidence that the nutraceutical supplement used in this project may represent a therapeutic strategy to reduce the impact of NSC on ID-prone horses.

### 4.2. Nutraceuticals

Several bioactive compounds have been studied as nutraceutical candidates for their potential therapeutic effect on IR in humans and rodents, and a few in horses [21,22,23,51,52,53,54]. Albeit, numerous ingredients have been suggested for mitigating IR in horses; most are based on data collected in humans and rodents and only a few have strong evidence in horses. In the current study, we selected a mixture of marine-derived omega-3 fatty acids, glutamine, brewer’s yeast, and vitamin E as these ingredients are well documented in human and rodent models to have a beneficial effect on insulin and glucose dynamics. Omega-3 fatty polyunsaturated fatty acids have been shown to increase insulin sensitivity and improve metabolic health in human and rodents through an improved mitochondrial function and energy metabolism as well a reduction in systemic inflammation [32]. In horses, supplementation with omega-3 fatty acids has been shown to reduce inflammatory mediators and modulate fat metabolism [55,56,57]. Although specific effects on insulin dynamics in insulin-sensitive horses are equivocal [55,58], studies in ID horses have shown improvements in their insulin sensitivity with marine-derived omega-3 fatty acid supplementation [26,59]. Plasma glutamine concentrations in humans have been shown to be inversely related to insulin resistance and supplementation improves the insulin sensitivity in rodent and human models [60]. The systemic administration and dietary supplementation of glutamine have shown to increase insulin sensitivity in the muscle and normalize hepatic glucose production in dietary-induced IR rodents [31,60]. An increased glutamine supply to the muscle of IR rodents downregulates the expression of proteins that regulate inflammation and decreases the expression of the adaptor protein GRB10, an inhibitor of insulin signaling [60]. Additionally, glutamine supplementation reduces adiposity and glucose uptake by adipose tissue, which may contribute to an overall improvement in insulin sensitivity in diet-induced IR [31]. While there have been several studies looking at glutamine supplementation in the context of mediating exercise-related oxidative stress and immune suppression [61,62], to the best of our knowledge, there are no data on its insulin sensitizing effect in horses. Similarly, vitamin E has been extensively studied in horses in relation to exercise and muscle-related benefits due to its antioxidant capacity [63,64]; however, no studies have looked at its direct effect on ID in horses. Studies in diabetic or overweight humans have shown vitamin E supplementation reduces fasting insulin and insulin sensitivity proxy measures. This effect is proposed to be mediated through the inhibition of glucose oxidation, reduction in oxidative stress, and enhancing the expression of adiponectin, which is known as an insulin-sensitizing adipokine [33,65]. Hypoadiponectinemia has been associated with ID and obesity in humans and horses [66,67] and, interestingly, pasture laminitis [68]. Lastly, multiple studies have investigated the effects of brewer’s yeast to improve insulin sensitivity, with the main mechanism of action associated with the fact that it contains a bioactive source of chromium, also known as a glucose-tolerant factor [28]. In turn, chromium’s suggested action is proposed to be through increased insulin receptor activation, resulting in enhanced cellular insulin signal transduction [69]. Chromium-rich yeast supplementation in humans has shown to improve glucose tolerance and insulin resistance [70]. Horses supplemented with chromium-yeast or chromium propionate tended to have lower postprandial glucose and insulin responses to a concentrate meal [29,71].

### 4.3. Dietary Starch Challenge Model

To evaluate the potential protective effect of the nutraceutical, we employed a carbohydrate (CHO) challenge model, in which 16 predisposed horses were subjected to a high-starch diet for four weeks. The selected horses had a history of having ID, were overweight, and some had a history of laminitic episodes. These horses had been strictly managed for their metabolic disease by housing in dry lots and limiting dietary NSC. It has been repeatedly shown in different models that the insulin sensitivity in horses adapted to NSC-rich diets decreases over time [12,14,72,73,74]. The mechanisms behind CHO-induced IR are associated with the desensitization of insulin receptors and intracellular insulin signal transduction due to repeated postprandial insulin surges [17,75,76]. Consequently, reduced peripheral insulin-stimulated glucose disposal leads to compensated hyperinsulinemia which may further exacerbate IR [7]. Four weeks of consuming an NSC-rich diet in the current study provoked a similar response as seen in previous studies. The consumption of 0.6 g NSC/kg BW/meal (NSC calculated as starch + ethanol soluble carbohydrates) twice daily reduced the peripheral insulin sensitivity of the placebo group (the glucose clearance rate decreased by ~40%, the time in the positive phase increased by ~100%, and the insulin concentrations at 75 min were increased by ~45%). Interestingly, the NSC load in this model (1.2 g NSC/kg BW/d from concentrate plus ~1.3 g NSC/kg BW/d estimated from hay) was much lower than that of previously used CHO models (~6–12 g NSC/kg BW/d [14,72,74]). This indicates that a daily intake of relatively small amounts of hydrolysable NSC is sufficient to induce IR in a short period of time in predisposed horses. This agrees with recent data, illustrating that the threshold for the dietary NSC intake per meal to avoid the exacerbation of hyperinsulinemia in ID horses is even lower (i.e., 0.1 g NSC per kg BW/meal) than previously suggested [18]. It should be noted that the previously described CHO models were often designed to trigger clinical laminitis. Conversely, in the current study, we wanted to impose a significant dietary challenge on the endocrine system while minimizing the risk for laminitis. This was successfully achieved, and no horses showed symptoms of a laminitic episode. Therefore, the current experimental NSC challenge offers a safe and clinically relevant model to evaluate the effect of CHO in ID-prone horses; in particular, when testing intervention strategies, as was the objective in the current study.

In contrast to the placebo group, the insulin sensitivity parameters did not change from the baseline measures in horses receiving the nutraceutical, indicating that the active ingredients in the supplement had a protective effect. Neither the NSC challenge, nor the nutraceutical intervention had any effect on the body weight or body condition score, indicating that the observed effects on insulin sensitivity were not caused by a change in the body composition. Basal insulin concentrations are commonly used by veterinarians to assess the insulin status. In the current study, both groups had basal plasma insulin concentrations of approximately 30 µIU/mL after 4 weeks of treatment (Figure 1). According to the most recent consensus statement on the diagnosing criteria for ID, these concentrations would define the horses as “suspected ID” [40]. Elevated resting insulin concentrations were not attributable to a postprandial effect since morning feed was withheld. The horses did have access to grass hay prior to sampling; however, the NSC concentrations of the hay were relatively low (i.e., 9.1%) and were not expected to have a considerable impact. Therefore, the observed basal hyperinsulinemia was likely the result of compensatory pancreatic insulin secretion and/or reduced hepatic insulin clearance associated with IR [77]. Basal insulin concentrations were not affected by treatment, indicating that the nutraceutical supplement did not mitigate basal insulin concentrations compared to the placebo. Basal glucose concentrations were not different between groups, which was expected as most ID horses are able to maintain euglycemia due to the pancreatic and hepatic compensatory mechanisms.

### 4.4. Hyperinsulinemia and Insulin Sensitivity

To assess the effects of the nutraceutical on tissue insulin sensitivity, a CGIT was performed before and after 4 weeks of supplementation. The insulin concentrations at >20 µIU/mL at 75 min and a duration of the positive phase > 45 min are the two most common parameters used to diagnose horses as having IR [37]. At week 4, the insulin concentrations were approximately 30 and 50 µIU/mL at t = 75 min for the nutraceutical and placebo group, respectively, indicating that both groups had IR. However, the plasma insulin concentrations at t = 75 min for horses receiving the nutraceutical was not different than their baseline concentrations, while those for the placebo group were still considerably elevated above the baseline at this timepoint, indicating much slower insulin clearance in the placebo group. This shortened period of hyperinsulinemia in the nutraceutical-treated horses suggests that supplementation could be beneficial in reducing the risk of laminitis development as prolonged hyperinsulinemia has been shown to experimentally induce laminitis [42,78].

After 4 weeks of supplementation, the blood glucose positive-phase glucose clearance time for horses receiving the nutraceutical was 32 min compared to 54 min for the placebo group (Figure 1), providing further evidence that the nutraceutical-supplemented horses were more insulin-sensitive. While no scientific threshold value has been determined for the glucose clearance rate during CGIT, this is perhaps the best indicator of IR as it clearly shows the rates of the insulin-mediated glucose uptake in response to a standardized dose of exogenous glucose and insulin. Using the CGIT in previous studies, we have recorded average glucose clearance rates of ~2.5 mg/dL/min for ID horses and ~5 mg/dL/min for insulin-sensitive horses [39]. Glucose clearance rates in the current study were 4.5 and 2.8 mg/dL/min for the nutraceutical and placebo group, respectively, illustrating that horses supplemented with the nutraceutical were able to clear blood glucose from circulation 61% faster compared to the placebo group. Based on previously reported glucose clearance rates in IR and non-IR horses, we can conclude that the placebo group had more IR than the nutraceutical group (Figure 1). Since glucose concentrations are directly linked to glucose clearance and the time in the positive phase, glucose concentrations at 45 min were significantly lower in the nutraceutical-supplemented horses compared to the placebo group. Moreover, the nutraceutical-supplemented horses were already below the baseline (i.e., in the negative phase) at that time, while the placebo horses were still in the positive phase. Consequently, Δ45 glucose concentrations were negative in the nutraceutical-supplemented group and positive in the placebo group.

### 4.5. Dietary Starch Challenge Model and Grass Pasture Non-Structural Carbohydrates

It has been reported that 46% of all laminitis cases result from grazing lush pasture [79]. The high incidence of pasture appears to be driven by the NSC content of the forage, as the prevalence of pasture-associated laminitis cases rise from 2 to 5% during the spring and summer months, coinciding with changes in the forage NSC content [16,80,81]. Despite these observations, a dietary starch challenge model was selected to control and quantify the starch intake during the nutraceutical supplementation of ID-susceptible horses on dry lots. Aside from the short duration of the challenge period, it could be argued that an apparent limitation of this model is that the NSC fraction of oat grain primarily consists of starch with lesser amounts of water-soluble carbohydrates, contrary to grasses which have large amounts of water-soluble carbohydrates plus fructans and lesser amounts of starch [19]. Whether there is a significant difference in the mechanisms by which different types of NSC induce laminitis is debatable, as both starch and WSC from pasture have shown to result in a significant rise in blood insulin concentrations, particularly in IR horses [16,82,83,84]. To provide some insight and relevance to pasture-managed horses, we compared the NSC intake (calculated as water soluble carbohydrates + starch to include fructans) from the current study with the estimated NSC intake of a horse grazing on pasture during early summer. The average 500 kg horse consumed approximately 1.7 kg NSC/d in the current study. The reported NSC values for cool-season grasses, which are the predominant species in most equine pastures, range from 95 to 188 g/kg DM [85,86]. This range is dependent on the species and season, with greater amounts in the spring and fall (170–188 g/kg DM) than in the summer months (95–100 g/kg DM). Estimates of the voluntary DM intake for grazing horses is 2–3% of their body weight per day [34]. Using this range to estimate the forage intake of the same 500 kg horse grazing on cool-season grass, the NSC intake would range between 1.0 and 1.5 kg/d during the summer months and 1.8 and 2.7 kg/per during the spring and fall seasons. The 1.7 kg/d of NSC supplied by our starch challenge model exceeds the expected NSC intake during summer grazing and is equivalent to the lower estimates of that during fall and spring. However, considering horses graze in 15–20 bouts during a 24 h period [87] and the fact that the horses in the current experiment consumed the starch diet within 1 h, the starch intake (~250 g/meal per 500 kg horse) alone would exceed the NSC intake per bout during spring and fall (120–180 g/bout). Although more work is needed before implementation, the above estimations suggest that the nutraceutical supplement could potentially mitigate the negative effects of seasonal changes in NSC in pasture grass on insulin sensitivity and hyperinsulinemia-associated laminitis in pastured horses.

## 5. Conclusions

In conclusion, this study shows that the dietary nutraceutical supplement containing a combination of glutamine, omega-3 fatty acids, vitamin E, and yeast reduced the progression of IR in predisposed horses during a 4-week high-NSC challenge. This CHO overload model can provide a safe and clinically relevant model to evaluate the effect of CHO in ID-prone horses.

## Figures and Tables

**Table 1:** Nutrient composition of the daily ration on dry matter basis.

Nutrient	Concentrate	Grass Hay
	% of DM	
DE (Mcal/kg)	3.4	2.07
Crude protein	16.5	10.1
Acid detergent fiber	9.1	36.8
Neutral detergent fiber	20.1	62.4
Water-soluble carbohydrates	7.8	11.2
Ethanol-soluble carbohydrates	6.4	6.9
Starch	35.1	2.2
Non-fiber Carbohydrates	53.8	17.5
Calcium	1.2	0.38
Phosphorus	1.18	0.34
Magnesium	0.22	0.17
Potassium	1	1.84
Sodium	0.286	0.017
	PPM	
Iron	158	201
Zinc	163	18
Copper	34	5
Manganese	109	112
Molybdenum	2	1.6

**Table 2:** Body weight, body condition score, age, and initial CGIT results.

Phenotypic Measure	Placebo	Nutraceutical	SEM	*p*-Values
Initial body weight (kg)	514.8	575.9	22.42	0.08
Delta body weight (kg) ^a^	18.1	16.1	3.07	0.65
Initial body condition score	6.5	7.2	0.52	0.41
Delta body condition score ^a^	−0.1	−0.4	0.24	0.27
Age (years) *	18.9	16.8	1.21	0.13
Initial CGIT measures				
Delta glucose conc. 45 min (mg/dL)	−18.5 ± 12.3	−21.1 ± 17.7	-	-
t = 75 insulin (µIU/mL)	36.5 ± 20.90	33.5 ± 18.83	-	-
Time in positive phase (min)	29.5 ± 6.48	30.2 ± 9.54	-	-
Glucose clearance rates (mg/dL/min)	4.5 ± 1.06	4.8 ± 1.61	-	-

^a^ Delta: post- minus pre-supplementation measurements. * Age was not known for all horses due to limited history on horse donations (*n* = 7 known for placebo, *n* = 6 known for nutraceutical group). CGIT: Combined glucose insulin tolerance test. Delta glucose concentrations: glucose concentrations at 45 min minus concentrations at baseline. Data are presented as least square means ± standard error of the mean with exception of the initial CGIT data which is presented for descriptive purposes as the average ± standard deviation since it was used as a covariate in later statistical analyses. N = 8 for each placebo and nutraceutical group.

**Table 3:** Digital pulse measurements over time.

	Week 1	Week 2	Week 3	Week 4		*p*-Values	
					Treat	Week	Treat*Week
Placebo	0.33 ± 0.11 ^a^	0.47 ± 0.15 ^b^	0.50 ± 0.20 ^b^	0.34 ± 0.20 ^ab^	0.85	0.006	0.32
Nutraceutical	0.16 ± 0.11 ^a^	0.47 ± 0.15 ^b^	0.61 ± 0.20 ^b^	0.56 ± 0.19 ^ab^			

^ab^ Main effect of week, where week 1 was different from weeks 2 and 3 (*p* ≤ 0.004). Data are presented as least square means ± standard error of the mean. N = 8 for placebo and nutraceutical group.

**Table 4:** Effect of nutraceutical or placebo treatment on CGIT parameters.

				*p*-Values		
	Placebo	Nutraceutical	SEM	Treat	Covariate	Independence of Covariate ^a^
Basal glucose conc. (mg/dL)	98.0	93.5	2.18	0.17	0.1	0.97
Basal insulin conc. (µIU/mL)	30.1 (17.33–52.33)	31.6 (21.03–47.48)	NA	0.90	0.06	0.86
Glucose 45 min conc. (mg/dL)	101.4	78.1	7.39	0.04	0.60	0.82
Delta 45 glucose conc. (mg/dL) *	3.5	−15.5	6.24	0.05	0.68	0.79
Insulin 75 min conc. (µIU/mL)	50.9	29.7	4.02	0.003	0.003	0.85
Glucose clearance rates in positive phase (mg/dL/min)	2.8	4.5	0.53	0.05	0.43	0.73
Positive-phase glucose AUC (mg/dL/min)	1889.3	1512.7	179.75	0.16	0.20	0.73
Time in positive phase (min)	53.7 (36.79–78.47)	32.1 (23.37–44.20)	NA	0.03	0.82	0.89
Nadir glucose conc. (mg/dL)	76.6	65.1	4.67	0.11	0.16	0.74
Time until nadir (min)	103.1	76.9	7.53	0.03	0.95	0.61

AUC, area under the curve; Treat, treatment; NA, not applicable. * Delta: t = 45 minus t = 0 min glucose concentrations. ^a^ Non-significant *p*-values indicate covariate was not affected by treatment and can be used in the model. Data are presented as least square means ± standard error of the mean. Basal insulin concentrations and time in positive phase data were log transformed; data are back-transformed means ± 95% CI.

## Data Availability

Dataset available on request from the corresponding author.
